# Chronic disease prevalence and associations in a cohort of Australian men: The Florey Adelaide Male Ageing Study (FAMAS)

**DOI:** 10.1186/1471-2458-8-261

**Published:** 2008-07-30

**Authors:** Sean A Martin, Matthew T Haren, Anne W Taylor, Sue M Middleton, Gary A Wittert, Members of the Florey Adelaide Male Ageing Study (FAMAS)

**Affiliations:** 1 Discipline of Medicine, Freemasons Foundation Centre for Men's Health, University of Adelaide, Adelaide, SA, 5000, Australia; 2 Spencer Gulf Rural Health School – Whyalla Campus, University of South Australia, Whyalla-Norrie, SA, 5608, Australia; 3 Population Research and Outcome Studies Unit, Department of Health, Government of South Australia, SA, 5000, Australia; 4 Department of Public Health, University of Adelaide, Adelaide, SA, 5000, Australia; 5 FAMAS (Florey Adelaide Male Aging Study), University of Adelaide, Adelaide, SA, Australia

## Abstract

**Background:**

An increasing proportion of Australia's chronic disease burden is carried by the ageing male. The aim of this study was to determine the prevalence of asthma, cancer, diabetes, angina and musculoskeletal conditions and their relationship to behavioural and socio-demographic factors in a cohort of Australian men.

**Methods:**

Self-reports of disease status were obtained from baseline clinic visits (August 2002 – July 2003 & July 2004 – May 2005) from 1195 randomly selected men, aged 35–80 years and living in the north-west regions of Adelaide. Initially, relative risks were assessed by regression against selected variables for each outcome. Where age-independent associations were observed with the relevant chronic disease, independent variables were fitted to customized multiadjusted models.

**Results:**

The prevalence of all conditions was moderately higher in comparison to national data for age-matched men. In particular, there was an unusually high rate of men with cancer. Multiadjusted analyses revealed age as a predictor of chronic conditions (type 2 diabetes mellitus, angina, cancer & osteoarthritis). A number of socio-demographic factors, independent of age, were associated with chronic disease, including: low income status (diabetes), separation/divorce (asthma), unemployment (cancer), high waist circumference (diabetes), elevated cholesterol (angina) and a family history of obesity (angina).

**Conclusion:**

Socio-demographic factors interact to determine disease status in this broadly representative group of Australian men. In addition to obesity and a positive personal and family history of disease, men who are socially disadvantaged (low income, unemployed, separated) should be specifically targeted by public health initiatives.

## Background

Almost four out of five Australians have at least one long-term health condition [1]. National expenditure on chronic disease and associated care now accounts for over two thirds of the entire health care budget [2]. Many of these diseases are preventable through the modification of the risk factors that contribute to their development [3]. In Australia, the National Chronic Disease Strategy (NCDS) has driven a renewed focus on the determinants and settings that promote the development of chronic conditions, particularly those that relate to the designated National Priority Areas of asthma, cancer, cardiovascular disease, diabetes and musculoskeletal conditions [4]. Much of this focus has centred on eliminating many of the health inequalities that persist in our population. To date however, the disproportionate disease burden carried by men, particularly the ageing male, has received little attention.

In Australia, as elsewhere, men display poorer health outcomes when compared with women [5,6]. The problems of male health in Australia reflect those increasingly noted in other developed nations. Men in Australia have a lower life expectancy than women (76.6 years compared with 82.0 years) with higher rates of mortality at all ages, a discrepancy that begins from birth [7]. There is a disproportionate level of chronic physical and psychological disease in Australian men [7] and higher rates of illness-related disability [1]. In addition, men display a higher prevalence of the major risk factors – smoking, lack of physical activity, poor nutrition and alcohol abuse – linked to the development of most major chronic diseases [3,8]. When combined with a reduced likelihood of adopting a healthy lifestyle [9] and a resistance to public health messages [10], the health and behaviour of ageing males in Australia should be an urgent concern.

Of the disease groups targeted by the NCDS, only musculoskeletal conditions have a lower prevalence in men at all ages as compared to Australian women [1] (with current indications that the incidence of rheumatoid- and osteo-arthritis in men aged over 60 is increasing faster than that of age-matched females [11]). Cardiovascular disease (CVD), the leading cause of death and disability in Australia, strikes more men than women across the entire age spectrum, with death rates among males aged 25–74 years two to three times that of females [12]. The rates of asthma in Australia are amongst the highest in the world and whilst the prevalence is greater in males than females during childhood, this reverses through the adult years. Men, however, aged 65 years and over in Australia are more likely to suffer disability and death as a result of their asthma [13]. Three times as many men as women over the age of 65 report some type of cancer [3], including a higher prevalence of those sex-independent cancers nominated as national priorities (colorectal cancer, lung cancer, melanoma, non-melanoma skin cancers and non-Hodgkin's lymphoma [1,14]). Men also present a significant challenge in the global diabetes epidemic, showing a higher prevalence of diabetes overall and an increased rate of mortality and complications arising from the condition [3,15].

Despite all these disparities, the health of men and the related changes in biological, psychological and social settings through ageing remains one of the most understudied areas of health research in Australia. Recognition of this is feeding a groundswell of support for men's health issues and policy initiatives in Australia, including a call by a number of peak and government bodies for a comprehensive men's health longitudinal study [16-18]. The Florey Adelaide Male Ageing Study (FAMAS) is a multi-disciplinary population cohort study of 1195 men, aged 35–80 years at recruitment and living in the north-west regions of Adelaide, Australia. We report here, in an analysis of the baseline cross-sectional data of the men in the cohort, the relationships between biological, social and demographic factors and the presence a number of chronic conditions considered to be of national priority.

## Methods

### The Florey Adelaide Male Ageing Study (FAMAS)

Details of the FAMAS design, procedures and participants have been published elsewhere [19]. Briefly, subjects were recruited at random from the Electronic White Pages (EWP) using six digits of the standard eight digit telephone number in addition to prefixes and exchanges for the North Western Suburbs of Adelaide. Randomly selected households were sent an introductory letter and brochure. Approximately two weeks later a call was made to the household and the male person aged between 35 and 80 years to last have his birthday was invited for a Computer-Assisted Telephone Interview (CATI) lasting no more than 15 minutes, together with an invitation to participate in the study. A series of questions relating to age, other socio-demographic variables, history of disease and presence of risk factors enabled comparison of responders and non-responders. Of those eligible to participate, 70.7% agreed to be interviewed (Participation Rate) and 45.1% ultimately attended a clinic (Final Response Rate). Non-responders were more likely to live alone, be current smokers, and had a higher prevalence of self-reported diabetes and stroke and lower prevalence of hypercholesterolemia. A comparison with 2001 Census data showed that participants matched the local and national population for most key demographics, but younger age groups and never married men under-represented and married men and elderly participants were over-represented [19].

Recruitment of participants occurred during two phases-from August 2002 to July 2003 and from June 2004 to May 2005 and complete clinical follow-up is scheduled to reoccur every five years from baseline throughout the life of the cohort. Follow-up questionnaires are designed by a multi-disciplinary investigative team using standard measures where available and are completed annually.

All protocols and procedures were approved by the Royal Adelaide Hospital Research Ethics committee and, where appropriate, the Aboriginal Health Research Ethics Committee of South Australia.

### Demographic and lifestyle measures

Demographic and lifestyle information were obtained by self-report questionnaires [19]. Socio-economic status was assessed using the relative advantage/disadvantage index from the Socio-Economic Indexes for Areas (SEIFA) by the Australian Bureau of Statistics. Higher scores indicate community-dwelling areas with a relatively high proportion of people with high incomes or a skilled workforce, as well as a relatively low proportion of people with low incomes and relatively few unskilled people in the workforce. Medical conditions were assessed by the following question item, 'Have you ever been told by a doctor that you have any of the following conditions?' Smoking status was determined using question items from recent Australian National Health Surveys [20]. Leisure-time physical activity was determined using items from the National Physical Activity Survey 1999 [21]. The semi-quantitative food frequency questionnaire (FFQ) developed by the Cancer Council of Victoria was used to estimate usual energy-providing macronutrient intakes [22].

### Anthropometric measures

Anthropometry was performed using standard protocols [23] in the morning, prior to breaking an overnight fast with participants barefoot and in light clothing. Height was measured using a wall-mounted stadiometer (Seca Model No. 220, Humberg, Germany). Body weight was obtained using digital platform scales (Wedderburn UW OFWB, Taiwan, CN) and maintained by on-site engineering services. Waist circumference was assessed using a fiberglass tape measure (Gulik II, Country Technology, Wisconsin, USA). Waist circumference was measured in triplicate, taken at the level of the narrowest point (or midway) between the lower costal border and the top of the iliac crest and read in the mid-axillary line, and the mean of the three measurements was used in analyses. Coefficients of variation for triplicate waist circumference measurements were less than 2.2% for 99.7% of the sample. Standard BMI cut-offs were used to classify participants in normal/underweight (<25 kg/m2), overweight (25–29.9 kg/m2), and obesity (≥ 30 kg/m2) categories.

### Hormone measures

Blood samples were drawn between 8 and 11 am after a 12-hour overnight fast. Fasting plasma glucose and lipids (triglyceride, total cholesterol, HDL, LDL) were measured on auto-analysers in the Diagnostic Services laboratory (IMVS) on a 24-hour basis. Determination of serum lipids was done enzymatically using a Hitachi 911 (Boehringer, Germany). The inter-assay CV's for the measurement of serum lipids are as follows; triglyceride 3%, total cholesterol 2.3%, HDL 6.7% and LDL 3.7%. Glucose was determined using an automated chemistry analyser system (Olympus AU5400, Olympus Optical Co Ltd. Japan). The inter-assay CV's for this assay are 2.5% at 3.5 mmol/L and 3.0% at 19.6 mmol/L. Glycated haemoglobin (HbA1c) was measured by high-pressure liquid chromatography (HPLC) using a spherical cation exchange gel (CV 2% at 6% of total haemoglobin).

### Chronic disease modelling

The conditions examined were based on those National Health Priority Areas covered in the recent National Chronic Disease Strategy [4], namely: cardiovascular disease, asthma, cancer, T2DM and musculoskeletal conditions. In the case of cardiovascular disease, angina was specifically selected for this model (as per [1]), given the significant burden of this condition in ageing men. The use of the term 'angina' refers to the presence (current or past) of any chronic stable angina or unstable angina as diagnosed by physician. Osteoarthritis and rheumatoid arthritis were chosen as representative of musculoskeletal conditions in this cohort, given the relatively low proportion of men with osteoporosis. Exposure variables were selected from established or suspected associations with the relevant outcome. Descriptive and tabular analysis was initially conducted to examine data distributions and observe patterns in exposure-outcome relationships.

Relative risks and 95% confidence intervals were estimated by binomial regression with the log link function using the GENMOD procedure in SAS (SAS Institute, Cary, NC). Firstly, the relative risk of each potential confounder on the outcome was examined to determine individual main effects. Next, bivariate regression models were fitted with the exposure variable and age to get age-adjusted relative risks. Age was then checked as a modifier for each of the exposure variables by fitting exposure, age and the age-exposure interaction. Age was considered a modifier for an exposure variable if the interaction had a p-value < 0.005 (to account for the multiple testing).

Those variables whose age adjusted relative risks had a *p *value < 0.25 were included in the final multivariate model [25]. This initial higher *p *value was selected to account for the confounding effect of age in the model (i.e. inclusion of age in any model of this type would decrease the overall probability of other variables reaching significance).

For all conditions, the socio-demographic variables investigated were: age, income, employment status, pension status, current smoker/ever smoked, physical activity, SEIFA (Index of Disadvantage), body mass index (BMI), and waist circumference. The choice of family history and co-morbidity variables varied according to outcome.

## Results

### Angina

Of the men examined, 6.5% (n = 78) reported to having been diagnosed with angina by a physician. The majority of cases were detected in men over 55 years (n = 60). Age was found to strongly associate with the condition, peaking in 55–64 year old men (Additional file 1). There was a reduced risk observed for men with higher gross household incomes (most notably in the $80, 000+ category), although this relationship was largely lost when controlled for age (Additional file 1). Widowed men were found to have an increased risk of angina when compared with men currently married or living with a partner, again this effect was not seen in the age-standardized model (Additional file 1). Not being in the workforce strongly increased the risk of developing angina in both models and those currently on a pension displayed an increased risk of angina (Additional file 1). When factored for the age of participants, there was a notable increase in the risk of angina for those with a family history of obesity and hypertension (although the latter was fractionally outside of the nominal significance range). There was a strong interaction observed between the presence of angina and other conditions. Men that had either diabetes, hypercholesterolaemia or hypertension all displayed an increased risk of also developing angina. With the exception of elevated cholesterol, this observation held when responses were age-adjusted (Additional file 1).

When all qualifying variables were included in the final model, age proved an extremely robust determinant of angina. When compared with men from the youngest bracket, the risk of developing angina near doubled in successive age groups (55–64 years: RR = 9.75 (3.35, 28.37); 65–80 years: RR = 18.79 (6.44, 54.79)). Having a family history of obesity also increased the likelihood of developing angina (RR = 1.84 (1.12, 3.01)). Again, a strong risk was observed if men were also hypercholesterolemic (RR = 2.97 (1.44, 6.10)) (Additional file 3).

### Asthma

The prevalence of men diagnosed with asthma in the cohort was 9.5% (n = 113). The majority of respondents (51.4%) with asthma were in the youngest age group (35–44 years).

A binomial regression model was applied to all selected exposure variables (Additional file. 1). When controlled for age, only waist circumference was found to associate with asthma. Being born in regions other than Australia or New Zealand, and being separated/divorced, were the only other factors nearing significance. All age-adjusted exposure variables with a p-value lower than 0.25, were included in the final model, in which the prevalence of asthma was associated with larger waist circumference (RR = 1.01 (1.00, 1.03)) and a history of separation/divorce (RR = 1.75 (1.18, 2.60)) (Fig. 3).

### Cancer

Within the cohort, 10.3% of men (n = 123) reported having some form of cancer. A variety of factors was shown to associate with cancer status following the application of regression models (Additional file 1). In the unadjusted binomial model, the strongest association was observed for age, with older age groups demonstrating increased cancer prevalence. Also, men not in the work force, currently receiving some form of pension and with hypertension were all found to have increased risk of all-types of cancer. When controlled for age, none of these associations held significance, with only men born outside of Australia/New Zealand showing a trend towards a lowered cancer risk (p = .03). Men from the highest SEIFA quartile were at increased risk of being diagnosed with some type of cancer, an observation that held after adjustment for age. In the converged model (Additional file 3), an increasing age (55–64 years: RR = 2.35 (1.24, 4.44); 65–80 years: RR = 3.88 (1.93, 7.79), and being unemployed (RR = 3.47 (1.29, 9.36), *p *= .014) were associated with cancer risk.

### Type 2 Diabetes Mellitus (T2DM)

Within the cohort, 15.6% of men (n = 187) either self-reported being diagnosed with T2DM or were diabetic by established clinical indicators (see Methods). There were numerous associations with diabetic status. Age showed a clear positive relationship with T2DM, with higher age groups showing elevated risk of disease. Income levels showed a particularly strong linear relationship with risk of T2DM, with lower risks observed for higher incomes in both the full and age-adjusted models. Being widowed was also shown to have a strong relationship with having T2DM in both models. Men who were either not in the work force or not on the pension displayed an increased risk of diabetes (although this was not significant for pensioners when adjusted for age). Current smokers tended to be less likely to be diabetic (although this was not observed in the age-adjusted model), whereas past smokers had an increased likelihood of diabetes. Obese participants showed an increased probability of T2DM, when compared to participants in normal weight ranges. In addition, participants with T2DM were also found to have larger waist circumferences. Men in the cohort who reported a family history of diabetes, showed a robust association with current T2DM status. As well, when controlled for age, those with a family history of obesity were moderately more likely to have T2DM. Finally, those diagnosed as hypertensive, were also at increased risk of T2DM (Additional file 2).

When all of the eligible effectors were entered in to the final model, there were numerous determinants of T2DM detected. There was a slight influence of age on diabetes observed in the cohort, with men aged above 55 tending towards an increased risk for the condition when compared with their younger counterparts. The overall trend of men with higher incomes showing a reduced possibility of T2DM neared significance. Widowed men were at greater risk of developing diabetes when compared with married/partnered men (RR = 1.91 (1.18, 3.08)). The waist circumference relationship observed initially was also conserved in the final model, with men with larger waists again showing an elevated risk of diabetes (RR = 1.02 (1.01, 1.04)). A family history of diabetes was again shown to strongly increase risk of having the condition (RR = 1.64 (1.21, 2.24)).

### Osteoarthritis & Rheumatoid Arthritis

The prevalence of the musculoskeletal conditions under consideration were 9.7% for osteoarthritis (n = 118) and 5.0% for rheumatoid arthritis (n = 60). In both cases, almost four out of five cases occurred in men over 55 years (77.6% for osteoarthritis and 76.7% for rheumatoid arthritis). There is a noted impact of age on the association with the selected exposure variables and these musculoskeletal conditions. When age was accounted for in the binomial models, there was a reduced risk for osteoarthritis in men born overseas and those with lower waist circumferences (Additional file 2).

The predictive model of osteoarthritis (Additional file 3) confirms age as a determinant of this condition, with the eldest age group showing a significantly increased risk (RR = 2.98 (1.49, 5.93)) (and a strong trend in the 55–64 age group). Of note, being obese (whilst showing a weak association in the binomial models) had a strong association with osteoarthritis in the final model (RR = 3.78 (1.35, 10.58)) (Additional file 3).

In the case of rheumatoid arthritis, none of the selected factors proved to be significant, although there was a slight tendency for such men to be unemployed (p = .028).

## Discussion

The prevalence of the chronic diseases examined in this study was generally similar to available estimates from national and local data sources. The higher cancer prevalence in comparison to age-matched men nationally was the noticeable exception, and this has previously been demonstrated in two other studies of the sampling area [24,25].

There was a clear effect of age on the conditions examined (with the possible exception of asthma) as has been shown previously in both men and women [26-28], however data from this study suggest that a number of social and demographic factors also contribute significantly to disease risk for each of the conditions studied, over and above age.

### Angina

The higher rate of mortality from cardiovascular disease is one of the major drivers of the life-expectancy gap between men and women. In this cohort, angina was reported by 6.5% of all participants, a similar prevalence to a previous study undertaken in the same region at approximately the same time (5.7% of participants aged 35+) [30], but higher than that reported in the 2004–5 NHS (4.2% of males 35–80) [1]. The high prevalence of obesity, T2DM and other risk factors for cardiovascular disease in this cohort may account for the discrepancy.

The effect of age was most noticeable in participants with angina, with men aged 65+ showing a markedly elevated risk of episodes of angina relative to younger men in accordance with observations of other population based studies [31,32].

Both hypertensive and diabetic men in the cohort showed an increased age-adjusted risk for angina. Likewise, elevated cholesterol was identified as a predictor of ever having angina in this multiadjusted model. All are well established risk factors for angina in men [1,3]. We also confirmed the independent relationship between obesity and angina. Zdrackovic (2007) in a twin registry study has recently pointed to a stronger genetic influence on the relationship between obesity and angina than previously reported [33]. Accordingly, a combination of family history of not only angina, but also obesity should prompt the assessment of and aggressive management of risk factors.

### Asthma

The prevalence of men with asthma in Australia (9.0% of the total population) is amongst the highest in the developed world. In this cohort, 9.4% of men reported having being ever diagnosed with asthma, with over half in the youngest age group. Whilst this prevalence is slightly higher than that observed in comparable studies of the region [29,34] and population surveillance data [1], it does provide limited support to recent observations of rising levels of asthma in ageing men in the face of overall decreases in other sub-groups [35]. It is suggested that this is, at least in part, related to the increase in obesity that has been observed in such men [3,36]. Asthmatic men in our study had higher waist circumferences in all level of analyses, independent of BMI. Such a finding is consistent with other epidemiological studies involving asthmatic men (see [37] for summary).

Whilst there have been numerous studies demonstrating a protective effect of marriage on developing asthma, to our knowledge this has been the first cohort study that has specifically shown an age-independent increase in asthma susceptibility for divorced or separated men. In an analysis of a group of middle aged British men, Ebrahim (1995) [38] speculated that the observed increase in asthmatic symptoms amongst recently separated men was most likely a combination of an increase in detrimental behaviour (alcohol consumption, smoking and physical inactivity) and the removal of spousal support. This suggestion is supported by recent data indicating that separated men aged 55+ are least likely to adhere to a written asthma plan [39].

The absence of any significant effect of smoking behaviour on asthma prevalence in this study is not in accordance with the common view of smoking being linked to adult-onset asthma. It is possible that smokers with airway disease had been classified by their treating practitioners as having chronic obstructive airways disease, and that those with asthma had been successfully convinced not to smoke. Also, there are a relatively high proportion of current smokers in the cohort who could be termed heavy consumers (i.e. greater than 25/day [40]). It has been suggested that such smokers, through a complex interaction of inherited and environmental factors may not display asthmatic-type symptoms [41].

### Cancer

The rising proportion of elderly men with cancer is leading to an increased investigation into the conditions which promote its development. Numerous demographic and behavioural factors have been found to associate with various types of cancer. A high proportion of men in this cohort had been diagnosed with some form of cancer when compared with age-matched men from local (4.1% of men aged over 35 years) and national surveillance data (3.3% of Australian men over 35 years). Whilst interpretation of this disparity is limited by the comparatively low number of participants in the study, a specialized health atlas of the region has identified that four out of the six highest Standardised Incidence Ratios for cancer are found in this study's catchment area [42]. This is currently the focus of intense investigation by local health authorities [43]. Interestingly we observed that age-matched men from the highest SEIFA quintile were at an increased risk of ever having cancer. In surveillance studies, lower SEIFA quintiles have generally been shown to associate with increased morbidity and mortality [44]. It has been argued that improvements in recent years in cancer screening and awareness may have created an artificially high impression of cancer prevalence amongst affluent American men [45]. Accordingly those men in areas of high social advantage within the study area (and better access to health services) may have an increased rate of cancer detection by physician. In contrast, men not currently employed, who have previously been shown to have a low rate of health care utilization [46,47], had one of the lowest rates of cancer in this study. It is tempting to speculate that these men may have cancers diagnosed late and therefore be prone to an increased cancer-related mortality.

### Type II diabetes mellitus (T2DM)

T2DM is a common condition of the older male. In this cohort, 15.6% of the men examined at clinic, had T2DM by either self-report or fasting glucose or HbA1c, a figure slightly higher than the prevalence reported by the AusDiab study (12.3% of men aged over 35 years [48]). Our data was collected 3 years later than AusDiab and the higher prevalence in our study most likely relates to the very high prevalence of obesity in the cohort [19].

In all levels of the analyses there were strong associations with the obesity (BMI, waist circumference, family history) in an order consistent with many other studies (see [49-51] for summary). One of the strongest associations observed with diabetes in this cohort, was the increased risk in widowed men. Following the loss of spousal support, widowed men (both middle aged and elderly) have been shown to exhibit poor self care, for example a reduction in physical activity and sub-optimal macronutrient intake [52]. Currently, there are some limited multi-disciplinary programmes for widowed men in Australia (support groups, 'tool-shed' social meetings etc.), which in part seek to demonstrate appropriate management of diabetes and appear to be having a positive effect on presenting complications [53].

There is also considerable evidence that supports the inverse association between income and T2DM, particularly in elderly men [54,55], as has been reported by others in both men and women in the same geographic region [56]. Whilst multifactorial in nature, a large portion of this effect appears to reside in the (perceived and real) expense of maintaining a well-balanced diet, and an over consumption of high fat/sugar foods at lower cost [57]. The other factors found to associate with diabetes in this study (namely, smoking and hypertension) were broadly consistent with other studies (see [58,59] for summary).

### Osteoarthritis & Rheumatoid Arthritis

The prevalence of musculo-skeletal conditions (particularly osteoarthritis, rheumatoid arthritis and osteoporosis) has been increasing in both in Australia [3] and abroad [60,61]. The proportion of elderly men with such conditions is also increasing, and to a greater extent than in ageing females [62]. In this study, the prevalence rates of ever-diagnosed osteoarthritis and rheumatoid arthritis for men in the sampling region was the same as that reported from national sources [3].

As expected, the strongest determinant of both osteoarthritis and rheumatoid arthritis was an increasing age [3]. Given the projected increased prevalence of these conditions in an ageing population and the associated impact on disability and public health costs, there is an urgent need for research identifying earlier markers and preventative treatments for these conditions. There is also a large body of evidence that identifies obesity as a risk factor for developing osteoarthritis [63-65]. Moreover once osteoarthritis is established, its progression and consequent limitations are more pronounced in obese men [66]. Whilst it was generally assumed that this effect is mediated through a decrease in physical activity [67], our findings are consistent with recent studies showing a minimal or non-existent relationship between osteoarthritis and physical activity patterns [68]. The mechanism by which obesity increases the risk of osteoarthritis is likely more than a simple mechanical effect [69]. The combined problem of an ageing and increasingly obese population and the morbidity and costs associated with osteoarthritis emphasise the importance of further study and primary prevention.

In a study of this type there are several limitations of the design that may limit findings. Firstly, the use of self-report data (whilst common to most health surveys and epidemiological studies) likely underestimates the true prevalence of any conditions, an effect repeatedly observed in male respondents [3]. Obviously, the type of self-report data used (current vs. 'ever-diagnosed by doctor' conditions) necessitates caution when comparing rates of disease amongst different sources. The prevalence of any condition can often differ substantially by region (even between local and statistical divisions). Indeed data from the latest National Health Survey advises standard errors of up to 25% for some conditions (e.g. angina, cancer) [1]. Second, the use of point-prevalence data in trying to examine associations with such dynamic diseases is not ideal, although this is common in most epidemiological studies of this type. Third, whilst the study included a wider range of variables than most, the complex nature of the conditions examined meant that much of the variability remains unexplained. Whilst including too many predictors can dilute an analysis of this type, inclusion of additional data might have further clarified factors associated with disease risk (e.g. alcohol consumption, nutrient, and energy intake). Finally, the directionality of many of the observed relationships cannot be determined in cross-sectional studies of this sort.

## Conclusions & implications

This analysis was designed to give an indication of the chronic disease burden in one of the few cohort studies specific to the ageing male worldwide, and the biological, behavioural and environmental settings that associate with these conditions. Whilst many proposals and strategies have been developed to address the problem of chronic disease and ageing populations, the unique challenges posed by elderly men still receives disproportionate attention.

This study demonstrated that a high proportion of men are currently suffering from chronic disease, including most of the conditions recently identified as National Health Priorities in Australia. It is clear from this study and others like it, that these conditions are further exacerbated with age. Furthermore, the prevalence of conditions examined in this study were largely equivalent to available data for age-matched men from the region, providing further qualified support to the representativeness of the cohort to the local population.

Obesity was associated with most of the diseases studied, and for some, having one or more obese parents added to the risk (angina, diabetes), even if those affected were not themselves obese, (at least at the time point examined). Many men had multiple, often related, conditions (angina, cancer, diabetes) and this multi-disease state is particularly prominent in elderly men [70], underscoring the importance of primary prevention. Apart from age and obesity a number of social and demographic factors were associated with chronic disease prevalence. Spousal support is demonstrably important; separated and widowed men have an increased prevalence of asthma and diabetes, respectively. Men from low-income households were at greater risk of diabetes. Participants not in the work force (most likely due to their disease) were shown to be more likely to have angina or cancer.

The ongoing collection of data from this cohort, with a high retention rate to date [19], will provide clearer information about cause and effect relationships. Since DNA has also been collected and stored, rapid advances in technology will permit an assessment of gene-environment interactions in the future.

The challenges that the ageing male presents in the current global fight against chronic disease have important policy and public health implications. Men still occupy the majority of the workforce, and in the background of an ageing population, understanding the many facets of the development of disease and disability are vital for any functional and productive society.

## Competing interests

The authors declare that they have no competing interests.

## Authors' contributions

GAW & MTH conceived of the study. SAM participated in the design, management and coordination of the study and drafted the manuscript. AWT provided crucial initial infrastructural support and contributes to the management of the study. GAW acts as Chief Investigator and continues to manage the study. MTH participated in the study design and coordination. SMM performed statistical analyses. All of the authors read and approved the final manuscript.

## Pre-publication history

The pre-publication history for this paper can be accessed here:



## Supplementary Material

Additional file 1 Table 1. Risk of angina, asthma and cancer by personal, behavioural and socioeconomic factors (attached). Click here for file

Additional file 2 Table 2. Risk of type 2 diabetes, osteoarthritis and rheumatoid arthritis by personal, behavioural and socioeconomic factors (attached). Click here for file

Additional file 3 Table 3. Multivariate model of personal, behavioural and socioeconomic predictors of selected chronic diseases (attached). Click here for file
