# Supplementary material for: Chronic disease prevalence and associations in a cohort of Australian men: The Florey Adelaide Male Ageing Study (FAMAS)
Source: BMC Public Health. 2008 Jul 30;8:261. doi: 10.1186/1471-2458-8-261 (PMC2531108; doi:10.1186/1471-2458-8-261)
Supplement: Additional file 1 — Table 1. Risk of angina, asthma and cancer by personal, behavioural and socioeconomic factors (attached). [file 1471-2458-8-261-S1.doc]

| **EXPOSURE VARIABLE** | **ANGINA**  **Unadj. RR (CI) Age-adj. RR (CI)** | | **ASTHMA**  **Unadj. RR (CI) Age-adj. RR (CI)** | | **CANCER**  **Unadj. RR (CI) Age-adj. RR (CI)** | |
| --- | --- | --- | --- | --- | --- | --- |
| **Age**  35-54  55-64  65-80 | Reference  10.64 (5.29,21.41)*****  4.78 (2.23,10.26)***** |  | Reference  1.08 (0.76, 1.54)  0.80 (0.54, 1.20) |  | Reference  2.27 (1.35, 3.81)*****  5.62 (3.64, 8.69)***** |  |
| **Income**  <$12 000  $12 001-$20 000  $20 001-$30 000  $30 001-$40 000  $40 001-$50 000  $50 001-$60 000  $60 001- $80 000  $80 000+ | Reference  0.93 (0.47, 1.84)  0.70 (0.34, 1.45)  0.54 (0.24, 1.23)  0.42 (0.18, 1.02)  0.20 (0.07, 0.61)*****  0.23 (0.08, 0.65)*****  0.11 (0.03, 0.39)***** | 0.80 (0.36, 1.80)  0.69 (0.30, 1.61)  0.87 (0.34, 2.27)  0.82 (0.30, 2.27)  0.47 (0.14 ,1.63)  0.58 (0.18 ,1.89)  0.30 (0.08, 1.20) | Reference  0.56 (0.29, 1.08)  0.80 (0.43, 1.46)  0.73 (0.38, 1.40)  0.84 (0.45, 1.57)  0.55 (0.28, 1.10)  0.59 (0.30, 1.14)  0.79 (0.44, 1.43) | 0.58 (0.30, 1.12)  0.79 (0.43, 1.45)  0.67 (0.34, 1.30)  0.76 (0.40, 1.44)  0.49 (0.24, 0.99)  0.52 (0.26, 1.03)  0.70 (0.37, 1.31) | Reference  1.60 (0.86, 2.97)  0.93 (0.47, 1.83)  0.93 (0.46, 1.88)  0.32 (0.12, 0.83)  0.55 (0.25, 1.22)  0.42 (0.18, 0.97)  0.37 (0.16, 0.84) | 1.43 (0.78, 2.60)  0.95 (0.49, 1.82)  1.44 (0.74, 2.83)  0.58 (0.22, 1.49)  1.15 (0.53, 2.53)  0.91 (0.40, 2.11)  0.91 (0.40, 2.10) |
| **Region of Birth**  Australia / NZ  Other | Reference  1.16 (0.75, 1.82) | 0.93 (0.57, 1.54) | Reference  0.65 (0.45, 0.92) | 0.65 (0.45,0.93) | Reference  0.83 (0.57, 1.20) | 0.67 (0.47, 0.96) |
| **Marital Status**  Married / Living with Partner  Separated / Divorced  Widowed  Never Married | Reference  0.59 (0.24, 1.45)  2.72 (1.27, 5.83)*****  0.49 (0.13, 1.99) | 0.65 (0.25, 1.70)  1.41 (0.53, 3.76)  1.13 (0.26, 5.01) | Reference  1.67 (1.12, 2.49)  1.60 (0.76, 3.36)  1.07 (0.50, 2.10) | 1.64 (1.10, 2.44)  1.78 (0.84, 3.77)  0.99 (0.48, 2.02) | Reference  0.83 (0.46, 1.49)  1.72 (0.82, 3.63)  0.47 (0.15, 1.45) | 0.94 (0.53, 1.68)  1.01 (0.50, 2.05)  0.83 (0.28, 2.47) |
| **Employment Status**  Employed  Unemployed  Not in Workforce | Reference  3.12 (0.75, 13.09)  7.40 (4.26, 12.86)***** | 2.92 (0.63, 3.49)  3.46 (1.61, 7.44)***** | Reference  1.51 (0.72, 3.19)  0.96 (0.69, 1.33) | 1.49 (0.70, 3.17)  1.18 (0.74, 1.89) | Reference  2.40 (0.91, 6.32)  3.73 (2.60, 5.37)***** | 2.24 (0.82, 6.14)  1.52 (0.87, 2.65) |
| **Pension Status**  No | 0.19 (0.12, 0.31)***** | 0.43 (0.23, 0.81) | 1.04 (0.76, 1.43) | 1.32 (0.89,1.98) | 3.19 (2.27, 4.49)***** | 1.43 (0.87, 2.36) |
| **Smoking**  Current Smoker  *Yes*  Ever Smoked  *Yes* | 1.44 (0.81, 2.57)  0.59 (0.36, 0.98) | 1.06 (0.60, 1.87)  1.52 (0.93, 2.48) | 0.93 (0.64, 1.34)  0.95 (0.69, 1.29) | 0.90 (0.62,1.31)  0.95 (0.69,1.30) | 0.63 (0.40, 1.01)  1.05 (0.74, 1.49) | 0.88 (0.56, 1.41)  0.97 (0.69, 1.36) |
| **Physical Activity**  Sedentary  Insufficient  Sufficient | Reference  0.79 (0.47, 1.35)  0.94 (0.55, 1.65) | 0.74 (0.45, 1.23)  0.78 (0.46, 1.32) | Reference  1.03 (0.71, 1.48)  0.81 (0.53, 1.24) | 1.04 (0.72,1.49)  0.82 (0.54,1.25) | Reference  0.63 (0.39, 1.02) 1.08 (0.74, 1.56) | 0.66 (0.41, 1.05)  0.97 (0.68, 1.39) |
| **BMI**  Underweight (<20)  Normal (20-24.99)  Overweight (25-30)  Obese (>30) | 1.54 (0.21, 1.14)  Reference  1.30 (0.67, 2.53)  1.72 (0.88, 3.38) | 1.90 (0.21, 7.15)  1.26 (0.61, 2.59)  2.00 (0.96, 4.20) | 1.26 (0.33, 4.77)  Reference  0.90 (0.59, 1.39)  1.43 (0.94, 2.18) | 1.28 (0.34, 4.84)  0.91 (0.59, 1.41)  1.42 (0.93, 2.17) | 0.65 (0.10, 4.47)  Reference  1.05 (0.69, 1.62)  0.75 (0.46, 1.24) | 0.69 (0.10, 4.91)  0.98 (0.65, 1.48)  0.76 (0.47, 1.23) |
| **SEIFA**  Quartile 1  Quartile 2  Quartile 3  Quartile 4 | Reference  0.54 (0.30, 0.99)  0.48 (0.22, 1.04)  0.98 (0.51, 1.88) | 0.59 (0.33, 1.05)  0.46 (0.21, 0.98)  0.93 (0.49, 1.74) | Reference  1.12 (0.78, 1.61)  0.94 (0.59, 1.50)  1.28 (0.80, 2.04) | 1.11 (0.77, 1.60)  0.93 (0.58, 1.49)  1.28 (0.80, 2.05) | Reference  1.21 (0.80, 1.84) 0.99 (0.58, 1.69)  1.95 (1.24, 3.09)***** | 1.88 (1.23, 2.87)  0.95 (0.57, 1.59)  1.30 (0.87, 1.95)* |
| **Waist** | 1.02 (1.00,1.04) | 1.02 (1.00, 1.04) | 1.02 (1.01, 1.03)***** | 1.02 (1.01, 1.03)***** | 1.00 (0.99, 1.02) | 1.00 (0.99, 1.02) |
| **Family History**  Diabetes  Obesity  Heart Attack  Stroke  Macrovascular  High BP  Prostate Cancer | 1.29 (0.79, 2.13)  1.61 (1.00, 2.61)  1.87 (1.10, 3.17)  1.46 (0.88, 2.42)  1.90 (1.01, 3.57)  1.90 (0.97, 3.75) | 1.61 (0.93. 2.79)  2.43 (1.40, 4.23)*****  1.87 (1.06, 3.33)  1.47 (0.85, 2.57)  1.90 (0.97, 3.75)  2.91 (1.36, 6.24) | 1.45 (1.04, 2.03)  0.87 (0.62, 1.21)  1.18 (0.81, 1.70) | 1.46 (1.04, 2.04)  0.86 (0.61, 1.20)  1.18 (0.81, 1.71) | 0.92 (0.63, 1.35)  0.78 (0.51, 1.18)  1.13 (0.77, 1.65)  1.11 (0.77, 1.59)  1.58 (0.99, 2.52)  1.00 (0.63, 1.60)  0.70 (0.42, 1.15) | 1.01 (0.70, 1.47)  0.91 (0.61, 1.37)  1.05 (0.72, 1.52)  1.09 (0.77, 1.55)  1.45 (0.92, 2.29)  1.14 (0.72, 1.81)  0.71 (0.44, 1.14) |
| **Conditions**  Diabetes  High Cholesterol  High BP | 3.34 (2.18, 5.13)*  2.48 (1.41, 4.37)*  2.44 (1.44, 4.13)* | 2.49 (1.41,4.41)*  1.24 (0.73, 2.10)  2.29 (1.31,3.98)* | 1.44 (1.00, 2.07)  1.13 (0.82, 1.56)  1.38 (1.00, 1.90) | 1.54 (1.05, 2.25)  1.13 (0.82, 1.57)  1.51 (1.07, 2.11) | 1.67 (1.13, 2.45)  0.98 (0.69, 1.38)  1.71 (1.18, 2.49)***** | 1.07 (0.73, 1.58)  0.92 (0.65, 1.28)  0.97 (0.66, 1.43) |

Data are taken from binomial regression of selected variables against each condition and expressed as relative risk (95% confidence intervals). Significance was taken at *p<*0.005

**Table 1. Risk of angina, asthma and cancer by personal, behavioural and socioeconomic factors.**
