# Supplementary material for: Chronic disease prevalence and associations in a cohort of Australian men: The Florey Adelaide Male Ageing Study (FAMAS)
Source: BMC Public Health. 2008 Jul 30;8:261. doi: 10.1186/1471-2458-8-261 (PMC2531108; doi:10.1186/1471-2458-8-261)
Supplement: Additional file 2 — Table 2. Risk of type 2 diabetes, osteoarthritis and rheumatoid arthritis by personal, behavioural and socioeconomic factors (attached). [file 1471-2458-8-261-S2.doc]

| **EXPOSURE VARIABLE** | **DIABETES**  **Unadj. RR (CI) Age-adj. RR (CI)** | | **OSTEOARTHRITIS**  **Unadj. RR (CI) Age-adj. RR (CI)** | | **RHEUMATOID ARTHRITIS**  **Unadj. RR (CI) Age-adj. RR (CI)** | |
| --- | --- | --- | --- | --- | --- | --- |
| **Age**  35-54  55-64  65+ | Reference  2.85 (1.97, 4.14)*****  4.25 (3.01, 5.99)***** |  | Reference  2.31 (1.42, 3.74)*****  4.15 (2.70, 6.38)***** |  | Reference  2.94 (1.51, 5.69)  3.64 (1.92, 6.82) |  |
| **Income**  <$12 000  $12 001-$20 000  $20 001-$30 000  $30 001-$40 000  $40 001-$50 000  $50 001-$60 000  $60 001- $80 000  $80 000+ | Reference  0.93 (0.47, 1.84)*****  0.70 (0.34, 1.45)*****  0.54 (0.24, 1.23)*****  0.42 (0.18, 1.02)*****  0.20 (0.07, 0.61)*****  0.23 (0.08, 0.65)*****  0.11 (0.03, 0.39)***** | 0.66 (0.46, 0.94)  0.44 (0.29, 0.67)*****  0.41 (0.24, 0.71)*****  0.38 (0.21, 0.68)*****  0.35 (0.19, 0.65)*****  0.55 (0.33 , 0.93)  0.44 (0.25, 0.78)***** | Reference  1.68 (0.84, 3.36)  1.19 (0.57, 2.45)  0.74 (0.32, 1.70)  0.65 (0.28, 1.54)  1.16 (0.55, 2.45)  0.34 (0.13, 0.93)  0.32 (0.12, 0.83) | 1.54 (0.77, 3.09)  1.20 (0.58, 2.51)  1.03 (0.44 ,2.44)  1.02 (0.42, 2.48)  2.01 (0.90, 4.53)  0.61 (0.21, 1.78)  0.64 (0.23, 1.78) | Reference  1.38 (0.57, 3.37)  0.73 (0.27, 1.97)  0.80 (0.29, 2.23)  0.39 (0.11, 1.35)  0.09 (0.01, 0.75)  0.43 (0.13, 1.36)  0.62 (0.23, 1.67) | 1.30 (0.54, 3.15)  0.73 (0.27, 1.99)  1.05 (0.37, 2.97)  0.54 (0.15, 1.94)  0.14 (0.02, 1.14)  0.67 (0.20, 2.23)  1.10 (0.38, 3.18) |
| **Region of Birth**  Australia / NZ  Other | Reference Class  1.43 (1.09, 1.86) | 1.19 (0.92, 1.54) | Reference  0.71 (0.48, 1.06) | 0.59 (0.40, 0.87) | Reference  1.12 (0.67, 1.88) | 0.94 (0.56, 1.58) |
| **Marital Status**  Married / Living with Partner  Separated / Divorced  Widowed  Never Married | Reference Class  0.82 (0.51, 1.33)  2.95 (1.97, 4.40)*****  0.77 (0.38, 1.57) | 0.90 (0.56, 1.43)  2.07 (1.41, 3.04)*****  1.30 (0.64, 2.65) | Reference  1.12 (0.66, 1.89)  1.52 (0.66, 3.49) 0.33 (0.08, 1.32) | 1.23 (0.72, 2.10)  0.99 (0.44, 2.21)  0.52 (0.13, 2.03) | Reference  1.13 (0.52, 2.44)  1.23 (0.31, 4.85)  1.01 (0.33, 3.16) | 1.21 (0.57, 2.59)  0.86 (0.22, 3.34)  1.57 (0.50, 4.98) |
| **Employment Status**  Employed  Unemployed  Not in Workforce | Reference  0.68 (0.17, 2.66)  3.11 (2.30, 4.08)***** | 0.60 (0.16, 2.34)  1.85 (1.27, 2.70)***** | Reference  2.04 (0.78, 5.31)  2.69 (1.89, 3.84)***** | 1.84 (0.67, 5.03)  1.23 (0.68, 2.21) | Reference  3.35 (1.05, 10.65)  3.12 (1.85, 5.27)***** | 2.90 (0.96, 8.77)  2.12 (1.00, 4.50) |
| **Pension Status**  No | 2.60 (2.00, 3.38)***** | 1.48 (1.06, 2.07) | 2.42 (1.72, 3.41)***** | 1.25 (0.76, 2.06) | 2.98 (1.80, 4.94)***** | 2.16 (1.15, 4.08) |
| **Smoking**  Current Smoker  *Yes*  Ever Smoked  *Yes* | 0.58 (0.39, 0.80)  1.76 (1.28, 2.41) | 0.76 (0.52, 1.12)  1.65 (1.21, 2.24) | 0.51 (0.30, 0.86)  1.53 (1.03, 2.26) | 0.90 (0.62, 1.31)  1.42 (0.97, 2.09) | 0.78 (0.41, 1.48)  1.63 (0.92, 2.89) | 0.99 (0.53, 1.86)    1.51 (0.85, 2.67) |
| **Physical Activity**  Sedentary  Insufficient  Sufficient | Reference  0.95 (0.69, 1.31)  0.96 (0.68, 1.36) | 0.95 (0.70, 1.30)  0.89 (0.63, 1.24) | 1.00 (0.66, 1.51)  Reference  0.86 (0.57, 1.30) | 1.02 (0.68, 1.53)  0.78 (0.52, 1.18) | 1.50 (0.86,2.60)  Reference  0.68 (0.35,1.33) | 1.50 (0.88 ,2.59)    0.62 (0.32, 1.22) |
| **BMI**  Underweight (<20)  Normal (20-24.99)  Overweight (25-30)  Obese (>30) | 0.73 (0.11, 5.02) Reference  1.37 (0.88, 2.13)  2.37 (1.54, 3.64)***** | 0.80 (0.12, 5.33)    1.28 (0.83, 1.96)  2.32 (1.53, 3.52)***** | 2.13 (0.54, 8.34)  Reference  1.40 (0.81, 2.39)  1.86 (1.08, 3.20) | 2.23 (0.56, 8.85)  1.32 (0.78, 2.25)  1.86 (1.09, 3.18) | 1.42 (0.20,10.13)  Reference  0.81 (0.41,1.60)  1.27 (0.65,2.48) | 1.49 (0.23, 9.66)    0.78 (0.39, 1.53)  1.25 (0.64, 2.43) |
| **SEIFA**  Quartile 1  Quartile 2  Quartile 3  Quartile 4 | Reference C  0.84 (0.61, 1.16)  0.68 (0.44, 1.04)  0.73 (0.45, 1.19) | 0.89 (0.65, 1.22)  0.65 (0.43, 0.99)  0.69 (0.43, 1.11) | Reference  0.83 (0.53, 1.31)  0.90 (0.53, 1.53)  1.62 (1.01, 2.60) | 0.88 (0.56, 1.37)  0.87 (0.52, 1.44)  1.57 (0.99, 2.49) | Reference  0.94 (0.51,1.74)  1.31 (0.69,2.51)  0.63 (0.23,1.75) | 0.98 (0.53, 1.80)  1.26 (0.66, 2.40)  0.61 (0.22, 1.70) |
| **Waist**  Continuous | 1.04 (1.03, 1.05)***** | 1.02 (1.01, 1.03)***** | 1.02 (1.01, 1.03)***** | 1.02 (1.01, 1.03)***** | 1.02 (1.00,1.04) | 1.01 (0.99, 1.04) |
| **Family History**  Diabetes  Obesity  Heart Attack  Stroke  Macrovascular  High BP  Prostate Cancer | 2.07 (1.55, 2.78)*****  1.37 (1.02, 1.83)  1.18 (0.88, 1.59)  0.84 (0.62, 1.15)  0.97 (0.71, 1.33)  1.09 (0.76, 1.54) | 2.27 (1.72, 3.01)*****  1.55 (1.17, 2.05)*****  1.08 (0.81, 1.43)  1.08 (0.81, 1.43)  0.88 (0.65, 1.19)  1.22 (0.87, 1.71) | 1.01 (0.68, 1.49)  1.45 (0.99, 2.15)  1.43 (0.98, 2.07)  1.68 (1.04, 2.71) | 1.14 (0.77, 1.67)  1.36 (0.93, 1.99)  1.41 (0.97, 2.03)  1.56 (0.98, 2.49) | 0.57 (0.32,1.04)  0.99 (0.56,1.75)  1.00 (0.59,1.70)  1.43 (0.82,2.49)  1.27 (0.68,2.38)  1.02 (0.50,2.08) | 0.61 (0.34, 1.10)  1.09 (0.62, 1.91)  0.94 (0.56, 1.59)  1.40 (0.81, 2.42)  1.18 (0.63, 2.21)  1.11 (0.55, 2.27) |
| **Conditions**  Diabetes  High Cholesterol  High BP | 1.25 (0.94, 1.67)  2.84 (2.01, 4.01)***** | 1.20 (0.91, 1.58)  1.92 (1.34, 2.75)***** | 1.44 (0.95, 2.17)  1.34 (0.92, 1.96)  1.83 (1.24, 2.70)***** | 0.99 (0.65, 1.49)  1.27 (0.87, 1.84)  1.19 (0.79, 1.78) | 1.20 (0.64,2.26)  0.76 (0.46,1.25)  2.24 (1.24,4.02) | 0.85 (0.43, 1.66)  0.72 (0.44, 1.17)  1.59 (0.86, 2.95) |

Data are taken from binomial regression of selected variables against each condition and expressed as relative risk (95% confidence intervals). Significance was taken at *p<*0.005

**Table 2. Risk of type 2 diabetes, osteoarthritis and rheumatoid arthritis by personal, behavioural and socioeconomic factors.**
