# Supplementary material for: Chronic disease prevalence and associations in a cohort of Australian men: The Florey Adelaide Male Ageing Study (FAMAS)
Source: BMC Public Health. 2008 Jul 30;8:261. doi: 10.1186/1471-2458-8-261 (PMC2531108; doi:10.1186/1471-2458-8-261)
Supplement: Additional file 3 — Table 3. Multivariate model of personal, behavioural and socioeconomic predictors of selected chronic diseases (attached). [file 1471-2458-8-261-S3.doc]

Table 3. Multivariate model of personal, behavioural and socioeconomic predictors of selected chronic diseases

| **PREDICTOR** | **ANGINA**  **Age-adj. RR (CI)** | **ASTHMA**  **Age-adj. RR (CI)** | **CANCER**  **Age-adj. RR (CI)** | **DIABETES**  **Age-adj. RR (CI)** | **RHEUMATOID ARTHRITIS**  **Age-adj. RR (CI)** | **OSTEOARTHRITIS**  **Age-adj. RR (CI)** |
| --- | --- | --- | --- | --- | --- | --- |
| **Age**  35-54  55-64  65+ | Reference  9.75 (3.35, 28.37)  18.79 (6.44, 54.79) ***** |  | Reference  2.35 (1.24, 4.44)*****  3.88 (1.93, 7.79)***** | Reference  1.84 (1.15, 2.95)  1.74 (0.98, 3.11) | Reference  2.01 (0.88, 4.61)  2.49 (0.95, 6.51) | Reference  1.93 (1.14, 3.25)  2.98 (1.49, 5.93)***** |
| **Income**  <$12 000  $12 001-$20 000  $20 001-$30 000  $30 001-$40 000  $40 001-$50 000  $50 001-$60 000  $60 001- $80 000  $80 000+ |  |  |  | Reference  0.66 (0.42, 1.04)  0.50 (0.30, 0.84)*****  0.53 (0.28, 0.98)  0.49 (0.24, 0.99)  0.45 (0.21, 0.96)  0.46 (0.23, 0.94)  0.56 (0.28, 1.15) |  | Reference  1.95 (0.90, 4.23)  1.82 (0.80, 4.13)  1.19 (0.45, 3.18)  1.16 (0.43, 3.17)  2.79 (1.13, 6.92)  0.81 (0.26, 2.54)  0.80 (0.25, 2.62) |
| **Region of Birth**  Australia / NZ  Other |  | Reference  0.70 (0.48, 1.03) | Reference  0.69 (0.44, 1.08) | Reference  1.25 (0.91, 1.71) |  | Reference  0.66 (0.44, 0.99) |
| **Marital Status**  Married / Living with Partner  Separated / Divorced  Widowed  Never Married |  | Reference  1.75 (1.18, 2.60)*****  0.98 (0.39, 2.45)  1.27 (0.62, 2.59) |  | Reference  0.86 (0.51, 1.47)  1.91 (1.18, 3.08)*****  0.95 (0.50, 1.80) |  |  |
| **Employment Status**  Employed  Unemployed  Not in Workforce |  |  | Reference  3.47 (1.29, 9.36)  2.00 (1.10, 3.65) | Reference  0.43 (0.08, 2.32) 1.49 (0.93 ,2.41) | Reference  3.38 (1.14, 9.99)  1.75 (0.79, 3.87) | Reference  2.50 (0.92, 6.77)  1.19 (0.66, 2.17) |
| **Smoking**  Current Smoker  *Yes*  Ever Smoked  *Yes* | 1.26 ( 0.73, 2.19) |  |  | 1.35 (0.95, 1.92) | 1.26 (0.67, 2.36) | 1.61 (0.88, 2.95)  1.02 (0.53, 1.96) |
| **Physical Activity**  Sedentary  Insufficient  Sufficient |  |  | 0.85 (0.50, 1.43)  Reference  1.03 (0.67, 1.59) |  | 1.57 (0.80, 3.05)  Reference  0.82 (0.39, 1.72) |  |
| **BMI**  Normal (20-24.99)  Underweight (<20)  Overweight (25-30)  Obese (>30) | Reference  1.52 (0.22, 10.26)  1.28 ( 0.57, 2.84)  1.52 (0.50, 4.58) |  |  | Reference  1.15 (0.22, 6.09)  1.00 (0.55, 1.81) 1.19 (0.59, 2.39) |  | Reference  1.38 (0.77, 2.47) 1.51 (0.72, 3.20)  3.78 (1.35, 10.58) |
| **SEIFA**  Quartile 1  Quartile 2  Quartile 3  Quartile 4 |  |  | Reference  1.21 (0.75, 1.95)  0.97 (0.53, 1.78)  1.63 (0.97, 2.73) |  |  |  |
| **Waist**  Continuous | 0.99 ( 0.96, 1.03) | 1.01 (1.00, 1.03)***** |  | 1.02 (1.01, 1.04)***** | 1.00 (0.97, 1.02) | 1.01 (0.99, 1.03) |
| **Family History**  Diabetes  Obesity  Heart Attack  Stroke  Macrovascular  High BP  Prostate Cancer | 1.84 (1.12, 3.01)*****  1.69 (0.85, 3.33) | 1.38 (0.97, 1.94) | 1.50 (0.90, 2.48)  0.70 (0.43, 1.14) | 1.64 (1.21, 2.24)*****  1.05 (0.75, 1.47) | 0.68 (0.38, 1.20)  1.41 (0.78, 2.53) | 1.54 (0.95, 2.47) |
| **Conditions**  Diabetes  High Cholesterol  High BP | 1.36 ( 0.80, 2.32)  2.97 (1.44, 6.10)*****  0.85 ( 0.46, 1.55) | 1.24 (0.83, 1.84)  1.21 (0.85, 1.72) |  | 1.16 (0.83, 1.60)  1.73 (1.10, 2.72) | 0.57 (0.33, 0.99)  1.00 (0.97, 1.02) | 1.21 (0.84, 1.75) |

Values are derived from binomial regression modelling and expressed as relative risk (95% confidence intervals). Predictors were included if age-adjusted *p* value <0.25. Significance was taken at *p*<0.01.
